# Comparitive effects of tetracyclines and citric acid on dentin root surface of periodontally involved human teeth: A scanning electron microscope study

**DOI:** 10.4103/0972-124X.44090

**Published:** 2008

**Authors:** Bhavya Shetty, Ashwini Dinesh, Hema Seshan

**Affiliations:** 1 *Senior Lecturer, Department of Periodontics, M.S. Ramaiah Dental College and Hospital, Bangalore – 560 054, India*; 2 *Reader, Department of Periodontics, M.S. Ramaiah Dental College and Hospital, Bangalore – 560 054, India*; 3 *Professor and HOD, Department of Periodontics, M.S. Ramaiah Dental College and Hospital, Bangalore – 560 054, India*

**Keywords:** Root surface conditioning, SEM root surface, SEM periodontitis

## Abstract

Periodontal diseases produce physical and chemical alterations in the root cementum. Various topical applications as root conditioning agents have been recommended as an adjunct to mechanical root surface debridement to remove smear layer, endotoxins and to expose collagen fibers on dentin surface. The objectives were to compare dentin surface changes following applications of tetracyclines and citric acid to the instrumented root surface of periodontally involved human teeth under scanning electron microscope.

The study group comprised of 80 dentin samples, which were prepared from periodontally-compromised teeth, planned for extraction. Diseased surfaces were root planed. The teeth were sectioned and solutions of tetracycline HCl, minocycline, doxycycline and citric acid were applied to the surfaces with cotton pellets for 5minutes.The root surface samples were then examined by scanning electron microscope.

Removal of smear layer in all the four groups was near total except a few areas. All four groups showed slight difference in mean number of total dentinal tubules. Minocycline and doxycycline showed no significant difference. The proportion of patent dentinal tubules was (74%) in tetracycline HCl group compared to minocycline (48.3%), doxycycline 42%), citric acid (52%), showing the differences statistically significant. Tetracycline group showed higher number of patent tubules when compared to minocycline, doxycycline and the difference was statistically significant.

Results of this study suggest that tetracycline is the best current tetracycline form for root surface conditioning as measured by its ability to affect both dentin smear layer removal and tubule exposure.

## INTRODUCTION

Regeneration of supporting tissue to tooth surfaces affected by periodontitis has long been an ideal of periodontal therapy. Periodontitis affected root surfaces are hypermineralized and contaminated with cytotoxic and other biologically active substances. Such surfaces are not biocompatible with adjacent periodontal cells, the proliferation of which is pivotal for periodontal wound healing. Madison and Hokett.[1]

An objective of periodontal treatment is the predictable regeneration of the periodontium in areas previously affected by periodontal disease. For regeneration to occur, it is necessary to eliminate calculus, bacterial plaque and other cytotoxic substances on or within the diseased root surface. Human root surfaces have been treated with many substances in an attempt to make the root physiologically acceptable for the regeneration of new connective tissue attachment. For over 90 years, various types of acids have been placed on root surfaces in attempts to modify the diseased tooth structure. Such treatment enlarges dentinal tubules into which healing connective tissue can enter.[2]

The rationale for periodontal therapy is aimed at elimination of periodontal disease, restoration of the periodontal tissues to a healthy, functional state and the subsequent maintenance of these tissues. The dental literature has clearly demonstrated that present modes of periodontal therapy are successful in achieving these goals; however, the ultimate goal of therapy is the regeneration of the attachment, which is lost during disease.[3]

Traditional surgical and non-surgical periodontal therapies aim at arresting periodontal disease by removal of plaque-“invested” tissues from disease-affected roots. However complete removal appears not possible with only mechanical debridment. Thus, root conditioning has been recommended as an adjunct to mechanical root surface debridment to remove smear layer and root associated endotoxins and to expose collagen fibers on the dentin surface.[4]

A number of agents have been proposed for the demineralization procedure which include EDTA, citric acid, minocycline, tetracycline, doxycycline, fibronectin phosphoric acid, Cohnns factor, sodium deoxycholate etc. These demineralizing agents when applied on the root surfaces remove the smear layer, eliminate the cytotoxic material like endotoxins, uncover and widen the orifices of dentinal tubules and expose the dentin collagen matrix. This collagen matrix is thought to provide a substrate which supports the chemotaxis, migration and attachm A number of agents have been proposed for the demineralization procedure which include EDTA, citric acid, minocycline, tetracycline, doxycycline, fibronectin phosphoric acid, Cohnns factor, sodium deoxycholate etc. These demineralizing agents when applied on the root surfaces remove the smear layer, eliminate the cytotoxic material like endotoxins, uncover and widen the orifices of dentinal tubules and expose the dentin collagen matrix. This collagen matrix is thought to provide a substrate which supports the chemotaxis, migration and attachment of those cells involved in wound healing and formation of new connective tissue attachment.

Invitro study on the effects of tetracycline HCl on dentin has revealed properties, which may be beneficial in periodontal regenerative therapy. Terranova *et al*, stated that root surface demineralization with tetracycline HCl enhanced soft tissue attachment, increase in fibronectin, an extra cellular matrix glycoprotein binding and enhanced fibroblast attachment and growth, while suppressing epithelial cell attachment and growth.[1] Furthermore, topical tetracycline HCl is adsorbed to and released from the dentin surface maintaining an antimicrobial property for at least fourteen days post therapy.

Minocycline has a low pH in concentrated solution, acts as a calcium chelator and its application results in enamel and root surface demineralization and removal of endotoxin invading untreated periodontally diseased roots.[5]

Citric acid has been shown to alter the surface characteristics of treated root surface by removing the smear layer, demineralises the planed surfaces and elutes bacterial endotoxins from the pathologically altered cementum surfaces.[6] Furthermore, citric acid demineralization of underlying dentin may enhance new connective tissue attachment by either accelerating the cementogenesis or by its bactericidal properties.

Doxycycline applied topically on root surfaces obtained from patients with periodontal disease invitro has shown high degree of substantivity and total exposure of dentin.[7]

Hence, in the present SEM study, an attempt has been made to compare the surface alteration on planed root surfaces following the application of tetracycline HCl , minocycline, doxyccyline, and citric acid.

## MATERIALS AND METHODS

In this study periodontally compromised teeth indicated for extraction were used, which fulfilled the following criteria. Patients between age group of 20-45 years.

No history of root planing, scaling or prophylaxis in the previous 6 months.

Attachment loss of about 50% - 60% Radiographic evidence of bone loss.

## PREPARATION OF THE SPECIMEN

The diseased periodontally affected mid root region of each tooth was chosen. A total of 80 dentin samples were prepared. All tooth cuts were made with a double-sided diamond disk in a slow-speed hand piece under copious water irrigation. The apical third of each root was removed and the remaining root was sectioned longitudinally through the root canal and cut horizontally to produce a 6 mm to 8 mm long tooth section. All pulpal tissue was thoroughly removed and an identification notch placed on the pulpal root surface. All teeth were rinsed and stored for at least 24 hours in 10% formalin. The dentin root surface was then instrumented by hand using a sharp Gracey 1-2 periodontal curette with 6 to 8 strokes per area to achieve a smooth glass-like surface.

A total of 80 specimens were prepared from extracted teeth, which were divided into 4 groups comprising of 20 specimens in each group.

1. Tetracycline HCl group: Dentin specimens treated with tetracycline (pH 1.8) for 5 min.
2. Minocycline group: Dentin specimens treated with minocycline (pH 3.8) for 5 min.
3. Doxycycline group: Dentin specimens treated with doxycycline (pH 2.2) for 5 min.
4. Citric acid group: Dentin specimens treated with citric acid (pH 1) for 5 min.

### Preparation of tetracycline solutions

Tetracycline Hcl (250 mg), doxycycline (100 mg) and minocycline (100 mg) solutions were prepared by adding a standard 1 ml of sterile water with the contents of one capsule. Each solution was thoroughly mixed in a sterile dappen dish until producing viscous slurry simulating clinical technique. The pH of each solution was tested using a pH meter.

### Preparation of citric acid solution

The saturated acid solution was made by slowly adding anhydrous citric acid to 50 ml of distilled water using a magnetic stirrer to mix the solution. Crystals were added until no more dissolved in solution and saturated to obtain citric acid at pH 1 which was checked using the pH meter.

### Application of the solutions

The dentin samples were lightly rubbed with 3 to 4 strokes with solution saturated cotton pellets that were changed every 30 seconds for a total period of 5 minutes to ensure consistent solution application. Following treatment, samples were rinsed with water for 20 seconds and air-dried.

## RESULTS AND DISSCUSSION

The nature of the periodontally exposed roots has been identified as one of the major factor influencing periodontal regeneration. Complex inflammatory, enzymatic and other biologic influences, which accompany periodontal diseases, produce physical or chemical alterations, which are particularly apparent in the root cementum. This periodontitis affected root surface may harbor bacterial cells and may be contaminated by endotoxins, which may repress fibroblast migration and proliferation on the cementum during the wound healing after the periodontal therapy. The diseased root surfaces loose the collagen fiber insertion into it. Also such surfaces may adsorb Ca, P, and F developing a highly calcified layer.[8]

Traditional treatment of pathologically altered root surfaces has relied on mechanical removal of plaque, calculus, root bound toxins and contaminated cementum and appears to be essential for periodontal regeneration. However it is not possible to completely decontaminate the root surface by mechanical therapy alone.[4]

Demineralization of root surfaces during periodontal therapy has been performed to enhance regeneration of the lost periodontal attachment. Demineralizing agents have shown to expose dentin collagen, widening the orifices of dentin tubules and cementum bound proteins. Furthermore, demineralizing agents have been found to elute retained toxins from the altered root surface. A number of agents have been proposed for the demineralization procedures including phosphoric acid, EDTA, citric acid and tetracycline[9]

Considering the above findings, an effort was made in this study to compare the surface characteristics of diseased tooth dentin after the application of tetracycline HCl, minocycline, doxycycline and citric acid by scanning electron microscopy.

In the present study, periodontally involved human teeth planned for extraction were used in the clinical simulation of various tetracycline forms and citric acid as root conditioning agents. Teeth with radiographic evidence of 50% or more loss of bone support, no history of scaling or root planing in past 12 months and attachment loss of at least 6mm.[5]

Total of 80 specimens were obtained from the roots of extracted teeth, which were categorized into four groups comprising of 20 specimens in each group.

The methods of application of root conditioners have varied among the clinicians that is placement of agent either passively or by burnishing. Though both the techniques were found to be equally effective in removing the smear layer in a study done by Roswen *et al*,[6] in the present study, passive application was preferred over burnishing technique as the latter may itself form smear layer which partially or completely obliterate the dentinal tubule openings.

Removal of smear layer in all the four experimental groups that is tetracycline HCl, minocycline, doxycycline and citric acid was near total except for few areas, which were covered by debris. This observation was consistent with that of Hanes *et al*,[10] Lafferty *et al*,[2] Madison and Hokett.[1]

All four groups showed slight difference in mean number of total dentinal tubules. Tetracycline HCl, minocycline and doxycycline showed slight difference in the mean number of tubules. Minocycline and doxycycline showed no significant difference in agreement with Madison and Hokett.[1] Tetracycline HCl and citric acid showed slight difference in the mean number of tubules. Lafferty[2] made similar observations.

The proportion of patent tubules to the number of dentinal tubules was (74%) in tetracycline HCl group compared to minocycline (48.3%), doxycycline (42.2%), citric acid (52.6%), showing the differences statistically significant. This may probably be attributed to the removal of smear layer to a lesser degree in citric acid, minocycline and doxycycline compared to tetracycline HCl owing to slightly lower pH of minocycline and doxycycline.

Tetracycline group showed higher number of patent tubules when compared to minocycline, doxycycline and the difference was statistically significant. This difference was probably due to the lower concentration of minocycline (pH 3.8), doxycycline (pH 2.2). Similar observations were made by Madison and Hokett[1] The increase in the tubule diameter was significantly greater for tetracycline group than citric acid group in this study.

In the present study it was established that root conditioning in all four experimental groups helped in removal of smear layer, exposure of dentinal tubules. Hence, their application as root conditioner have a significant role in periodontal wound healing and future new attachment *in vivo*.

The results of the present study are limited to physical findings of root surface changes and do not present in-vivo differences that may result from the physiologic effect of these root conditioning agents. Difference between our results and those of other studies may be related to the disease status of dentin specimen utilized, the concentration, time and mode of application of demineralizing agent or a combination of these variables. Hence, additional studies of these variables are needed.

A total number of 80 specimens were prepared from extracted teeth, which were divided into 4 groups comprising of 20 specimens in each group.

**Experimental A:**

Tetracycline HCl: Dentin specimen treated with tetracycline HCl (pH 1.8) for 5 minutes.

**Experimental B:**

Minocycline: Dentin specimen treated with minocycline (pH 3.8) for 5 minutes.

**Experimental C:**

Doxycyline: Dentin specimen treated with doxycycline (pH 2.2) for 5 minutes.

**Experimental D:**

Citric acid: Dentin specimen treated with citric acid (pH 1) for 5 minutes.

The processed dentin specimen for SEM examination and the microphotographs of representative areas were obtained at magnification ×3500.

At ×3500 magnification, the total number of dentinal tubules, the number of patent dentinal tubules and the proportion of patent tubules to the total number of dentinal tubules were calculated. The mean diameter of dentinal tubules was measured under the same magnification.

## EXPERIMENTAL GROUPS

### Experimental A: (Tetracycline HCl)

At ×3500 magnification, the total number of dentinal tubules, the number of patent dentinal tubules and the proportion of patent dentinal tubules to the total number of dentinal tubules were calculated and the mean diameter of dentinal tubule was measured.

**Figure 1:**
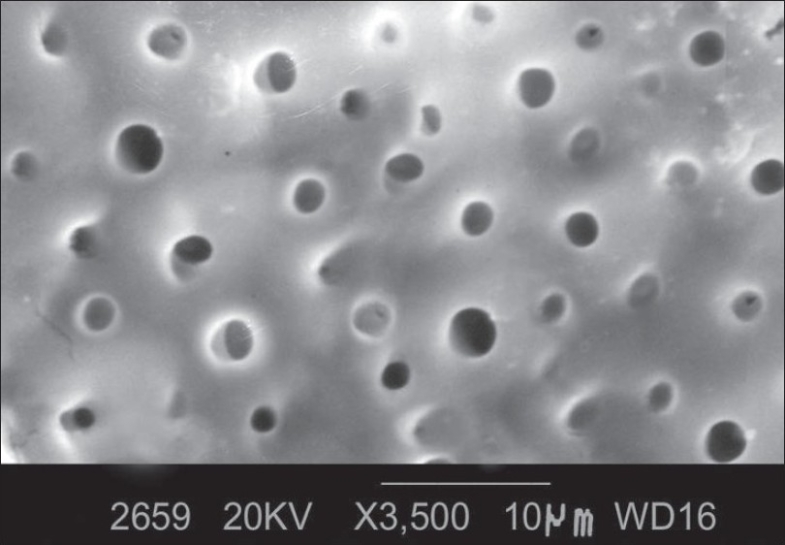
Morphology of root surface treated with tetracycline HCl at ×3500

The following observations were made [Figure 1]:

Smear layer was removed.

Orifices of dentinal tubules were clearly observed.

Most of the dentinal tubular orifices were patent and oval in shape.

Some dentinal tubules were occluded.

The number of dentinal tubules in each specimen was counted and total number of dentinal tubules in 20 specimens amounted to 630 with a mean 31.5±3.10 [Tables 1 and 2].

**Table 1:** Dentin tubules and patent dentin tubules in four experimental groups

Specimen no.	Tetracycline HCL			Minocycline			Doxycycline			Citric acid		
				
	No. of tubules	No. of patent tubules	%	No. of tubules	No. of patent tubules	%	No. of tubules	No. of patent tubules	%	No. of tubules	No. of patent tubules	%
1	30	24	80	20	12	60	22	12	54.5	29	15	51.7
2	28	18	64.2	28	10	35.7	24	12	50	34	20	58.8
3	32	26	81.2	23	11	47.8	20	12	60	32	14	43.7
4	32	20	62.5	26	12	46.1	25	10	40	30	16	53.3
5	38	29	76.3	20	10	50	26	08	30.7	27	13	48.1
6	40	34	85	24	10	41.6	20	08	40	32	22	68.7
7	34	27	79.4	20	09	45	20	09	45	32	16	50
8	30	22	73.3	30	08	26.6	24	04	16.6	27	11	40.7
9	32	22	68.7	26	10	38.4	20	06	30	26	14	53.8
10	32	20	62.5	24	10	41.6	24	12	50	30	18	60
11	30	24	80	22	12	54.5	26	08	30.7	28	18	64.2
12	30	26	86.6	20	12	60	24	10	41.6	29	14	48.2
13	28	20	71.4	24	11	45.8	28	10	35.7	30	16	53.3
14	30	24	80	20	16	80	20	12	60	32	15	46.8
15	28	18	64.2	20	10	50	22	14	63.6	28	16	57.1
16	30	20	66.6	20	10	50	24	08	33.3	24	14	58.3
17	34	22	64.7	26	10	38.4	20	08	40	22	12	54.5
18	30	26	86.6	22	11	50	23	10	43.4	20	13	65
19	32	26	81.2	20	12	60	20	10	50	30	20	66.6
20	30	20	66.6	20	14	70	20	08	40	28	21	75
Total	630	468	74.2	455	220	48.3	452	191	42.2	570	300	52.6

The Excel and SPSS (SPSS Inc, Chicago) software, student ‘t’, Chi-square test of significance (proportion)

**Table 2:** Mean total number of tubules in four experimental groups

Groups	No. of specimen	Total no. of dentinal tubules	Mean ± SD	Diff. between groups	
				
				Groups compared	Significance
I Tetracycline HCl	20	630	31.5 ± 3.10	I and II	*P* < 0.05
II Minocycline	20	455	22.75 ± 3.13	I and III	*P* < 0.05
III Doxycycline	20	452	22.6 ± 2.54	I and IV	*P* < 0.05
IV Citric acid	20	570	28.5 ± 3.52	II and III	*P* > 0.05
				II and IV	*P* < 0.05
				III and IV	*P* < 0.05

The Excel and SPSS (SPSS Inc, Chicago) software, student ‘t’, Chi-square test of significance (proportion)

The number of patent dentinal tubules in each specimen was counted and the total number of patent dentinal tubules in 20 specimen amounted to 468 with a mean 23.4±4.02 [Tables 1 and 4].

**Table 3:** Number of patent tubules in four experimental groups

Groups	Total no. of dentinal	No. patent dentinal tubules	Proportion of patent dentinal tubules	Difference between groups	
				
				Groups compared	Significance
I Tetracycline HCl	630	468	74.2%	I and II	*P* < 0.05
II Minocycline	455	220	48.3%	I and III	*P* < 0.05
III Doxycycline	452	191	42.2%	I and IV	*P* < 0.05
IV Citric acid	570	300	52.6%	II and III	*P* > 0.05
				II and IV	*P* > 0.05
				III and IV	*P* < 0.05

The Excel and SPSS (SPSS Inc, Chicago) software, student ‘t’, Chi-sequare test of sognificance (proportion)

**Table 4:** Mean number of patent tubules in four experimental groups

Groups	No. of specimen	No. of patent dentinal tubules	Mean no. of patent tubules ± SD	Difference between groups	
				
				Groups compared	Significance
I Tetracycline HCl	20	468	23.4 ± 4.02	I and II	*P* < 0.05
II Minocycline	20	220	11 ± 1.78	I and III	*P* < 0.05
III Doxycycline	20	191	9.55 ± 2.39	I and IV	*P* < 0.05
IV Citric acid	20	300	15.9 ± 3.60	II and III	*P* < 0.05
				II and IV	*P* < 0.05
				III and IV	*P* < 0.05

The Excel and SPSS (SPSS Inc, Chicago) software, student ‘t’, Chi-square test of significance (proportion)

The proportion of the number of patent dentinal tubules to the total number of dentinal tubules was 74.2% [Table 1].

The diameter of dentinal tubules was measured in each specimen [Table 5] and the mean tubular diameter of 20 specimen was calculated as 1.47µm with range of 1.30–1.58µm (±0.02) [Table 6].

**Table 5:** Size (diameter, μm) of patent dentinal tubules

Specimane no.	Tetracycline HCL		Minocycline		Doxycycline		Citric acid	
				
	No. of patent dentinal tubules	Mean ± SD	No. of patent dentinal tubules	Mean ± SD	No. of patent dentinal tubules	Mean ± SD	No. of patent dentinal tubules	Mean ± SD
1	24	1.43 ± 0.01	12	1.12 ± 0.01	12	1.15 ± 0.05	15	1.35 ± 0.02
2	18	1.47 ± 0.02	10	1.12 ± 0.00	12	1.12 ± 0.01	20	1.35 ± 0.01
3	26	1.47 ± 0.01	11	1.13 ± 0.01	12	1.11 ± 0.02	14	1.30 ± 0.04
4	20	1.45 ± 0.04	12	1.17 ± 0.05	10	1.11±0.015	16	1.38 ± 0.03
5	29	1.47 ± 0.02	10	1.13 ± 0.01	08	1.11 ± 0.01	13	1.35 ±0.02
6	34	1.50 ± 0.03	10	1.13 ± 0.01	08	1.11 ± 0.01	22	1.34 ± 0.05
7	27	1.49 ± 0.04	09	1.16 ± 0.05	09	1.11 ± 0.01	16	1.31 ± 0.06
8	22	1.49 ± 0.12	08	1.14 ± 0.03	04	1.11 ± 0.02	11	1.31 ± 0.06
9	22	1.49 ± 0.05	10	1.12 ± 0.01	06	1.11 ± 0.02	14	1.29 ± 0.05
10	20	1.48 ± 0.06	10	1.16 ± 0.05	12	1.12 ± 0.02	18	1.32 ± 0.04
11	24	1.44 ± 0.01	12	1.13 ± 0.01	08	1.11 ± 0.02	18	1.35 ± 0.02
12	26	1.44 ± 0.02	12	1.15 ± 0.02	10	1.11 ± 0.01	14	1.34 ± 0.01
13	20	1.45 ± 0.01	11	1.16 ± 0.02	10	1.11 ± 0.02	16	1.36 ± 0.01
14	24	1.45 ± 0.03	16	1.15 ± 0.03	12	1.10 ± 0.02	15	1.33 ± 0.04
15	18	1.46 ± 0.01	10	1.14 ± 0.01	14	1.10 ± 0.02	16	1.34 ± 0.04
16	20	1.47 ± 0.01	10	1.14 ± 0.01	08	1.11 ± 0.02	14	1.35 ± 0.03
17	22	1.47 ± 0.03	10	1.13 ± 0.02	08	1.12 ± 0.05	12	1.34 ± 0.03
18	26	1.48 ± 0.04	11	1.14 ± 0.03	10	1.11 ± 0.02	13	1.33 ± 0.02
19	26	1.47 ± 0.05	12	1.12 ± 0.01	10	1.11 ± 0.02	20	1.34 ± 0.02
20	20	1.48 ± 0.04	14	1.14 ± 0.03	08	1.13 ± 0.01	21	1.34 ± 0.02
Total	468	1.47 ± 0.02	220	1.14 ± 0.02	191	1.11 ± 0.01	300	1.33 ± 0.021

The Excel and SPSS (SPSS Inc, Chicago) software, student ‘t’, Chi-square test of significance (proportion)

**Table 6:** Comparision of mean diameter of dential tubules (patent) between different groups

Groups	No. of patent tubules	Size (diameter, μm)		Difference between groups	
			
		Range	Mean ± SD	Groups compared	Significance
I Tetracycline HCl	468	1.30 − 1.58	1.47± 0.02	I vs II	*P* < 0.05
II Minocycline	220	1.10 − 1.25	1.14 ± 0.02	I vs III	*P* < 0.05
III Doxycycline	191	1.07 − 1.24	1.11 ± 0.01	I vs IV	*P* < 0.05
IV Citric acid	300	1.23 − 1.42	1.33 ± 0.02	II vs III	*P* > 0.05
				II vs IV	*P* < 0.05
				III vs IV	*P* < 0.05

The Excel and (SPSS Inc, Chicago) software, student ‘t’, Chi-square test of significance (proportion)

### Experimental B: (Minocycline)

**Figure 2:**
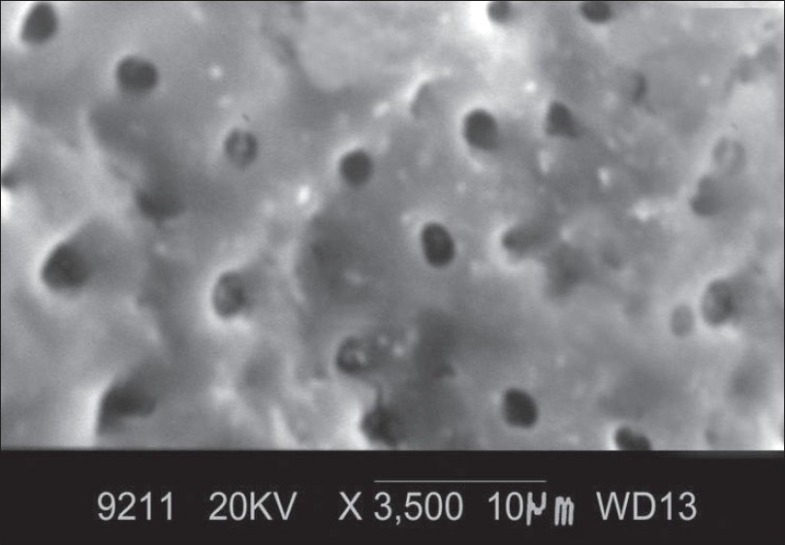
Morphology of root surface treated with minocycline at ×3500

At ×3500 magnification, the total number of dentinal tubules, the number of patent dentinal tubules and the proportion of patent dentinal tubules to the total number of dentinal tubules were calculated and the mean diameter of dentinal tubule was measured. The following observations were made [Figure 2]:

Smear layer was not completely removed.

Orifices of dentinal tubules were clearly seen.

Some of the dentinal tubular orifices were patent

Most of the dentinal tubular orifices were occluded.

The number of dentinal tubules in each specimen was counted and total number of dentinal tubules in 20 specimens amounted to 455 with a mean 22.75±3.13 [Tables 1 and 2].

The number of patent dentinal tubules in each specimen was counted and the total number of patent dentinal tubules in the 20 specimen amounted to 220 with a mean 11±1.78 [Tables 1 and 4].

The proportion of the number of patent dentinal tubules to the total number of dentinal tubules was 48.3% [Table 1].

The diameter of dentinal tubules was measured in each specimen [Table 5] and the mean tubular as 1.14µm with range of 1.10 – 1.25µm (±0.02) [Table 6].

### Experimental C: (Doxycycline)

**Figure 3:**
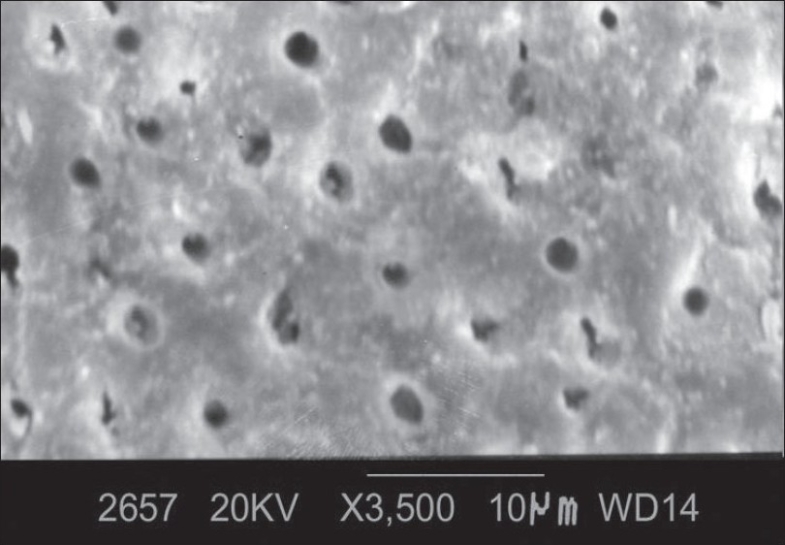
Morphology of root surface treated with doxycycline at ×3500

At ×3500 magnification, the total number of dentinal tubules, the number of patent dentinal tubules and the proportion of patent dentinal tubules to the total number of dentinal tubules were calculated and the mean diameter of dentinal tubule was measured. The following observations were made [Figure 3].

Smear layer was not completely removed

Orifices of the dentinal tubules were clearly seen.

Some of the dentinal tubular orifices were patent and oval in shape.

Some of the dentinal tubular orifices were occluded.

The number of dentinal tubules in each specimen was counted and total number of dentinal tubules in 20 specimens amounted to 452 with a mean 22.6±2.54 [Tables 1 and 2].

The number of patent dentinal tubules in each specimen was counted and the total number of patent dentinal tubules in the 20 specimen amounted to 191 with a mean 9.55±2.39 [Tables 1 and 4].

The proportion of the number of patent dentinal tubules to the total number of dentinal tubules was 42.2% [Table 1].

The diameter of dentinal tubules was measured in each specimen [Table 5] and the mean tubular diameter of 20 specimens was calculated as 1.11µm with range of 1.07 – 1.24µm (±0.01) [Table 6].

### Experimental D: Citric acid

**Figure 4:**
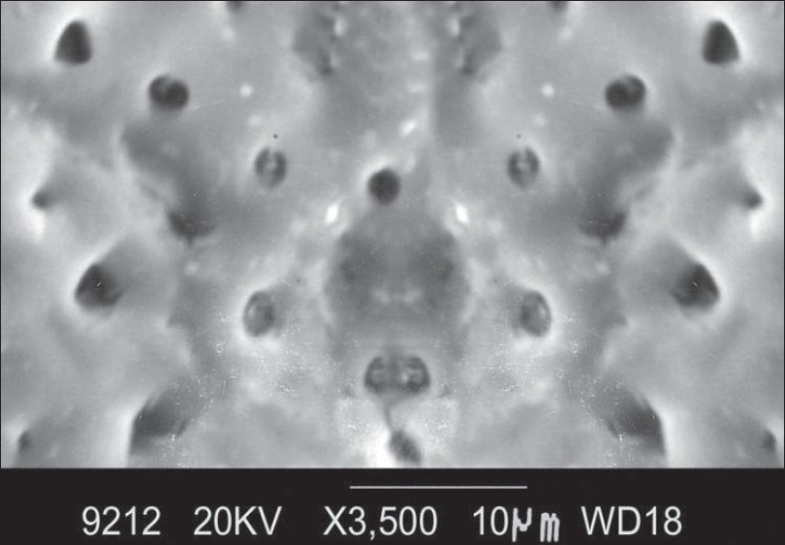
Morphology of root surface treated with citric acid at ×3500

At ×3500 magnification, the total number of dentinal tubules, the number of patent dentinal tubules and the proportion of patent dentinal tubules to the total number of dentinal tubules were calculated and the mean diameter of dentinal tubule was measured. The following observations were made [Figure 4].

Smear layer removed except for occasional areas.

Orifices of the dentinal tubules were clearly seen.

Many of the dentinal tubular orifices were patent and oval in shape.

Some of the dentinal tubular orifices were occluded

The number of dentinal tubules in each specimen was counted and total number of dentinal tubules in 20 specimens amounted to 570 with a mean 28.5±3.52 [Tables 1 and 2].

The number of patent dentinal tubules in each specimen was counted and the total number of patent dentinal tubules in the 20 specimen amounted to 300 with a mean 15.9±3.06 [Tables 1 and 4].

The proportion of the number of patent dentinal tubules to the total number of dentinal tubules was 52.6% [Table 1].

The diameter of dentinal tubules was measured in each specimen [Table 5] and the mean tubular diameter of 20 specimens was calculated as 1.33µm with range of 1.23 – 1.42µm (±0.02) [Table 6].

Total number of dentinal tubules, number of patent tubule, proportion of patent dentinal tubule to the total number of dentinal tubules and the mean diameter of dentinal tubules were considered. Significant difference between the groups were observed.

The Excel and SPSS (SPSS Inc, Chicago) software packages were used for data entry and analysis. Student “t” test was used to determine whether there was a statistical difference between the experimental groups. Proportions were compared using Chi-square test of significance.

## COMPARISION OF TETRACYCLINE HCL, MINOCYCLINE, DOXYCYCLINE, AND CITRIC ACID GROUPS

### Tetracycline HCl group (Exp. A) and Minocycline group (Exp. B)

The mean number of dentinal tubules in tetracycline HCl group was 31.5 ± 3.10 [Table 2] whereas in minocycline group it was 22.75 ± 3.13 [Table 2]. The mean difference in total dentinal tubules between Exp. Group-A and Exp. Group-B was statistically significant (*P*<0.05).

The mean number of patent dentinal tubule in tetracycline HCl group was 23.4 ± 4.02 [Table 4] whereas in Minocycline group it was 11±1.78 [Table 4], the difference between the two groups was statistically significant (*P*<0.05).

The proportion of patent tubules to the total number of dentinal tubules in tetracycline HCl group was 74.2% [Table 3]. When compared to minocycline 48.3% [Table 3] the mean difference was statistically significant (*P*<0.05).

The mean diameter of dentinal tubule in tetracycline HCl group was 1.47 ± 0.02 [Table 6]. When compared to Minocycline 1.14 ± 0.02 [Table 6] the difference was statistically significant (*P*<0.05).

### Tetracycline HCl group (Exp. A) and Doxycycline group (Exp. C)

The mean number of total dentinal tubules in tetracycline HCl group was 31.5 ±3.10 [Table 2] whereas in Doxycycline group it was 22.75 ± 3.13 [Table 2]. The mean difference in total dentinal tubules between Exp. Group-A and Exp. Group-C was statistically significant. (*P*< 0.05). The mean number of patent dentinal tubules in tetracycline HCl group was 23.4 ± 4.02 [Table 4] whereas in Doxycycline group it was 9.55 ± 2.39 [Table 4], the difference between the two groups was statistically significant (*P*<0.05).

The proportion of patent tubules to the total number of dentinal tubules tetracycline HCl group was 74.2% [Table 3]. When compared to Doxycycline group 42.2% [Table 3], the mean difference between the two groups was statistically significant (*P*<0.05).

The mean diameter of dentinal tubule in tetracycline HCl group was 1.47 ± 0.02 [Table 6]. When compared to Doxycycline 1.11 ± 0.01 [Table 6] the difference was statistically significant (*P*<0.05).

### Tetracycline HCl Group (Exp. A) and Citric acid group (Exp. D)

The mean number of total dentinal tubules in Tetracycline group was 31.5 ± 3.10 [Table 2] whereas in citric acid group it was 28.5 ± 3.52 [Table 2], the mean difference in total dentinal tubules between Exp. Group-A and Exp. Group-D was statistically significant (*P*<0.05).

The mean number of patent dentinal tubules in tetracycline HCl group was 23.4 ± 4.02 [Table 4] whereas in citric acid group it was 15.9±3.06 [Table 4], the difference between the two groups was statistically (*P*<0.05).

The proportion of patent tubules to the total number of dentinal tubules tetracycline HCl group was 74.2% [Table 3]. When compared with citric acid 42.2%, the mean difference was statistically significant (*P*<0.05).

The mean diameter of dentinal tubule in tetracycline HCl group was 1.47 ± 0.02 [Table 6]. When compared with citric acid 1.33 ± 0.02 [Table 6], the mean difference was statistically significant (*P*<0.05).

### Minocycline Group (Exp. B) and Doxycycline group (Exp. C)

The mean number of dentinal tubules in Minocycline group was 22.75 ± 3.13 [Table 2]. When compared with Doxycycline group 22.6 ± 2.54 [Table 2], the mean difference was statistically not significant (*P*>0.05).

The mean number of patent tubules in Minocycline groups was 11± 1.78 [Table 4]. When compared with Doxycycline group 9.55 ± 2.39 [Table 4], the difference between the two groups was statistically significant (*P*< 0.05).

The proportion of patent tubules to the total number of dentinal tubules in Minocycline group was 48.3% [Table 3]. When compared with Doxycycline group 42.2% [Table 3], the mean difference was statistically not significant (*P*>0.05).

The mean diameter of dentinal tubules in Minocycline group was 1.14 ± 0.02 [Table 6]. When compared with Doxycycline group 1.11 ± 0.01 [Table 6], the difference was statistically not significant (*P*>0.05).

### Minocycline Group (Exp. B) and citric acid group (Exp. D)

The mean number of total dentinal tubules in Minocycline group was 22.75 ± 3.13 [Table 2] whereas in Citric acid group it was 28.5 ± 3.52 [Table 2]. The mean difference in total dentinal tubules between Exp. Group-B and Exp. Group-D was statistically not significant (*P*>0.05).

The mean patent dentinal tubules in Minocycline group were 11 ± 1.78 [Table 4], whereas in citric acid group it was 15.9 ±3.06 [Table 4], the difference between the two groups was statistically significant (*P*< 0.05).

The proportion of patent tubules to the total number of dentinal tubules in Minocycline group was 48.3% [Table 3]. When compared with Citric acid 52.6% [Table 3], the mean difference was not statistically significant (*P*>0.05).

The mean diameter of dentinal tubule in Minocycline group was 1.14 ± 0.02 [Table 6]. When compared with Citric acid 1.33 ± 0.02 [Table 6] the difference was statistically significant (*P*<0.05).

### Doxycycline group (Exp. C) and Citric acid group (Exp. D)

The mean number of total dentinal tubules in Doxycycline group was 22.6 ± 2.54 [Table 2] whereas in Citric acid group it was 28.5 ± 3.52 [Table 2]. The mean difference in total dentinal tubules between Exp. Group-C and Exp. Group-D was statistically significant (*P*<0.05).

The mean patent dentinal tubules in Doxycycline group was 9.55 ± 2.39 [Table 4] whereas in Citric acid group it was 15.9 ± 3.06 [Table 4], the difference between the two groups was statistically significant (*P*<0.05).

The proportion of patent tubules to the total number of dentinal tubules in Doxycycline group was 42.2% [Table 3]. When compared with Citric acid 52.6% [Table 3], the mean difference was statistically significant (*P*<0.05)

The mean diameter of dentinal tubule in Doxycycline group was 1.11± 0.01 [Table 6]. When compared with Citric acid 1.33 ± 0.002 [Table 6] was statistically significant (*P*<0.05).

### CONCLUSION

A total of 80 specimens were obtained from extracted teeth, which were divided into four groups comprising of 20 specimens in each group. The root conditioning groups included four experimental groups-tetracycline HCl, minocycline, doxycycline and citric acid. The dentin root surface was instrumented by hand using curettes and 7×3mm dentin slabs were obtained. The root conditioning agents were applied for 5 minutes with cotton pellets. These specimens were subjected to SEM analysis.

Depending on the results, the following conclusions were drawn: All the four experimental groups were effective in removing the smear layer. The efficacy of smear layer removal was better and consistent in tetracycline group compared to minocycline, doxycycline and citric acid.

The exposure and widening of the dentinal tubules was noted in all four groups. The total number of dentinal tubules was statistically significant in tetracycline HCl followed by citric acid, minocycline and doxycycline.

The number of patent tubules found in tetracycline HCl was statistically significant than minocycline and doxycycline. However citric acid was more effective than minocycline and doxycycline.

The proportions of tubule to the total number of tubules were also statistically significant in tetracyclines than other groups. However citric acid showed higher efficacy than minocycline and doxycycline.

Tetracycline HCl group showed higher mean diameter of patent dentinal tubules. However citric acid showed higher values of mean diameter compared to minocycline and doxycycline.

Root surface conditioning by topical application of acidic solutions has been demonstrated to remove the smear layer and also any remaining root surface contaminants. It uncovers and widens the orifices of dentin tubules and unmasks the dentin collagen matrix thereby providing a substrate which supports chemotaxis, migration and proliferation of cells involved in periodontal healing and formation of new connective tissue attachment.

In the present study, though all four groups showed promise and efficacy, tetracycline HCl was found to be a better root conditioner.
